# Gamma-Glutamyltransferase: A Predictive Biomarker of Cellular Antioxidant Inadequacy and Disease Risk

**DOI:** 10.1155/2015/818570

**Published:** 2015-10-12

**Authors:** Gerald Koenig, Stephanie Seneff

**Affiliations:** ^1^Health-e-Iron, LLC, 2800 Waymaker Way, No. 12, Austin, TX 78746, USA; ^2^Iron Disorders Institute, Greenville, SC 29615, USA; ^3^Computer Science and Artificial Intelligence Laboratory, MIT, Cambridge, MA 02139, USA

## Abstract

Gamma-glutamyltransferase (GGT) is a well-established serum marker for alcohol-related liver disease. However, GGT's predictive utility applies well beyond liver disease: elevated GGT is linked to increased risk to a multitude of diseases and conditions, including cardiovascular disease, diabetes, metabolic syndrome (MetS), and all-cause mortality. The literature from multiple population groups worldwide consistently shows strong predictive power for GGT, even across different gender and ethnic categories. Here, we examine the relationship of GGT to other serum markers such as serum ferritin (SF) levels, and we suggest a link to exposure to environmental and endogenous toxins, resulting in oxidative and nitrosative stress. We observe a general upward trend in population levels of GGT over time, particularly in the US and Korea. Since the late 1970s, both GGT and incident MetS and its related disorders have risen in virtual lockstep. GGT is an early predictive marker for atherosclerosis, heart failure, arterial stiffness and plaque, gestational diabetes, and various liver diseases, including viral hepatitis, other infectious diseases, and several life-threatening cancers. We review literature both from the medical sciences and from life insurance industries demonstrating that serum GGT is a superior marker for future disease risk, when compared against multiple other known mortality risk factors.

## 1. Introduction

A comprehensive review by Whitfield in 2001 [1] described GGT in its traditional role as a marker of liver dysfunction, bile duct conditions, and alcohol consumption. Some generalized or summary medical and scientific literature still describe GGT in those terms [2]. However, Whitfield had already extended that description to include elevated GGT in association with risk of coronary heart disease, type-II diabetes (T2D), and stroke [1]. Although gamma-glutamyl compounds include antioxidants, inflammatory molecules, drug metabolites, and neuroreactive compounds [3], the major function of GGT is enabling metabolism of glutathione and glutathionylated xenobiotics. However, elevated GGT levels, as noted by Whitfield and others, contribute to prooxidant activity, particularly in the presence of iron or copper [4, 5]. When GGT levels are elevated, damage to red blood cell membranes can occur causing the release of these potentially toxic transition metals, which can further result in chain, prooxidant reactions [6]. Increased levels of prooxidation can lead to downstream cell, tissue, and DNA damage caused by oxidative and nitrosative stress and the generation of deleterious reactive oxygen species or nitric oxide (ROS or NO) [7]. This combination of factors is observed with increasing frequency in many chronic diseases. Other investigators have added many newly identified GGT-related diseases and conditions to a rapidly growing list that very recently was modified by Sreeram et al. [8] to even include GGT as a marker for oxidative stress in periodontal disease.

In this paper, we review an extensive research literature on GGT as a marker for disease. Not only do we demonstrate strong potential for GGT to predict later disease risk, but we also show variations in GGT levels among different population groups (gender, ethnic, and regional) and show evidence of temporal upward trends in population level GGT values, particularly in Korea and the United States. We believe this is indicative of both increased disease risk over time at the population level and evidence of changing environmental factors, such as excessive iron intake and increased exposure to xenobiotics, and nutritionally related impairment of cell membranes that may be playing a role. We hypothesize that GGT is a marker for glutathione depletion in the liver and that elevations in GGT reflect increased exposure to organic xenobiotics that are metabolized in the liver through glutathionylation. We suggest simple, primarily dietary lifestyle changes and targeted therapies that could lower GGT levels and improve health status across the population.

In the remainder of this paper, we will first present in Section 2 evidence linking elevations in GGT levels to cardiovascular disease risks, heart failure, cancer, and all-cause mortalities.  Section 3 presents correlations between GGT and antioxidant depletion linked to vascular impairment and introduces evidence linking environmental toxins to increased GGT and depleted antioxidants.  Section 4 provides literature support for the strong predictive power of GGT for obesity, liver diseases, MetS, prehypertension, and insulin resistance and evidence of a synergistic predictive relationship between GGT and serum ferritin (SF) levels as a marker of body iron stores.  Section 5 discusses GGT's predictive utility for gestational diabetes, a potential link to high SF, insulin resistance, and iron overload in pregnancy, and the associated risks.  Section 6 shows that the life insurance industry is well aware of the predictive power of GGT for mortality risk and uses this information effectively to inform insurance decisions.  Section 7 discusses ethnic differences observed in oxidative stress and iron measures and gives an indication that a rising tide of GGT levels among disproportionately affected populations might portend adverse health consequences.  Section 8 shows that GGT is a predictor for chronic kidney disease and albuminuria. Finally, we summarize our findings in a concluding section.

## 2. GGT in MetS, Heart Disease, Cancer, and Mortality

The first American epidemiologic study to test GGT levels was the Framingham Offspring Study (FOS). Between 1978 and 1982, 3,853 participants in the second phase of FOS were evaluated for most of the standard heart disease risk factors, including body mass index (BMI), alcohol consumption, smoking, blood pressure, lipids, liver enzymes (including GGT), fasting blood glucose, serum creatinine, C-reactive protein (CRP), and the presence of diabetes. Participants were followed up for a total of 20 years, with an interim assessment after 8 years to ascertain incidence of MetS. In 2007, Lee et al. [9] reported that baseline measures of GGT were significantly related to the onset of MetS by the end of the 8-year interim period. The relationship of GGT to MetS followed a dose-response relationship across each of participant quartiles 2 through 4, when compared to the first GGT quartile. Baseline peak GGT measures in the first quartile were 6 U/L for women and 11 U/L for men. After adjusting for age, gender, and alcohol use, and compared to participants in the first GGT quartile, the hazard ratios (HRs) for new onset MetS for men and women combined were 1.46 in quartile two, 1.83 in quartile three, and 2.54 in quartile four. The HRs were significant to *P* ≤ 0.001 for quartiles three and four, and *P* < 0.05 for quartile two. At the end of the full follow-up period (mean of 19 years), a total of 968 participants developed MetS and 535 incident cardiovascular disease (CVD), and 362 died. After multiple adjustments for standard CVD risk factors, participants in the highest GGT quartile experienced a 67% increase in CVD incidence (fully adjusted HR 1.67 (95% CI; 1.25 to 2.22)).

In 2010, Dhingra et al. [10] reported GGT-related heart failure outcomes in the same FOS cohort (mean age 44.5 years at baseline) who were followed up to the end of the entire study period (mean 23.6 years). Their report noted that 188 participants (111 men) developed new onset heart failure. Participants having baseline GGT levels above gender medians (16 U/L and 9 U/L, respectively, for men and women) experienced a 1.71-fold risk of heart failure compared to participants having GGT below their gender's baseline median. Importantly, each of these FOS studies showed that testing individuals a single time for GGT provided an* early* predictive marker for metabolic diseases, including MetS (sometimes referred to as insulin resistance syndrome), CVD, and heart failure, and that that prediction was valid well in advance of participants reaching these hazardous thresholds. This is important because GGT can be lowered by dietary means alone [11–13], or by making nominal dietary adjustments combined with minimally intrusive procedures, such as blood donation or therapeutic phlebotomy [14].

In 2007, Kazemi-Shirazi et al. [15] reported mortality outcomes for 283,483 attendees of the Vienna General Hospital for whom GGT tests were obtained as part of a routine test panel. Participants were monitored for up to 13 years and outcomes were matched against a national mortality database, a procedure that verified the distribution of causes of death, and gender- and age-matched death rates. This validation process indicated that study outcomes were comparable to those observed for the Austrian population. The investigators stratified outcomes based on age and gender. They established separate reference groups for men and women; each gender group was comprised of participants representing the lowest mortality risk category GGT. Combined, these gender-based cohorts represented approximately 37 percent of the entire study population. The upper GGT range limit for each reference group was 14 U/L for men and 9 U/L for women. Participants having GGT above those levels were grouped into four additional risk categories. For example, the next 30 percent of the total population (approximately 85,000 people) was comprised of women having GGT levels between 9 U/L and 17 U/L and men between 14 U/L and 27 U/L. Participants in this second category experienced a 20 percent increased risk of all-cause mortality. Mortality HRs stepped up in increments across each of three higher GGT risk categories. Consistent with the dose-response relationship often observed for GGT, the greatest mortality risk categories were for men and women having the highest GGT levels at baseline. Individuals in this high-risk category, representing 15 percent of the entire study group (GGT levels above 56 U/L for men and 36 U/L for women), experienced a 100 percent increased risk of mortality. Increased mortality risk in this highest GGT group expressed as a percentage across the most common causes of death was 130% for cancer mortality; 60% for both vascular mortality and death due to ischemic heart disease; and 40% for stroke deaths. Among liver disease and liver cancer deaths, hepatobiliary mortality and death due to hepatoma ranked the highest with HRs of 15.1 to 1 and 18.5 to 1, respectively. One notable ancillary finding of this large study population was that the increase in mortality risk was highest among participants under the age of 30 years when tested.

Similar to the above mortality results from Austria, a 2006-reported study covering 28,838 Finnish men and women [16] noted that GGT-related HRs were higher among individuals under age 60, both for nonfatal heart attack and fatal coronary heart disease (CHD). Among study cohorts, the lowest GGT-related risk quartiles were less than 16 U/L for men and 10 U/L for women. Above these reference levels, increasing GGT levels translated to increased risks for each outcome studied and reached statistical significance for CHD mortality among men with GGT levels above the male median level of 24 U/L. This was noted for each of the two models tested. In model one, after adjusting for age, study year, and study location, the HR for CHD mortality was 1.37 (95% CI; 1.06–1.78) for men with GGT between 16 and 24 U/L (second quartile GGT); 1.73 (95% CI; 1.34–2.24) for men with third quartile GGT (24–38 U/L); 2.06 (95% CI; 1.53–2.77) for GGT between 38 and 64 U/L; and 2.58 (95% CI; 1.86–3.58) for men in the 90th and above percentile GGT levels, or GGT above 64 U/L; all *P* trends were *P* < 0.01. For women, using the same model, CHD mortality risk reached a significant HR of 1.65 (95% CI; 1.07–2.54) for women in the first 15 percentiles of fourth quartile female GGT (20–32 U/L), also *P* < 0.01. Interestingly, threshold GGT levels at which higher CHD mortality risk became significant for both men and women were GGT (16–24 U/L) for men in the second GGT quartile and GGT (20–32 U/L) for women in the first section of their fourth GGT quartile. The lower initial HR for men compared to women (1.37 versus 1.65) was likely a factor of the lower absolute GGT levels (by 4 GGT U/Ls) in which significant risk was reached by men. However as expected, there were many more men than women in higher risk categories.

In a 2009-reported study based on data from the third US National Health and Nutrition Examination Survey, 1988–1994 (NHANES III), Ruhl and Everhart [17] studied mortality data through December 31, 2000. The data covered 14,950 adult NHANES participants. Investigators reviewed study-entry data obtained between 1988 and 1994 for both serum alanine aminotransferase (ALT) and serum GGT. ALT is a commonly measured liver enzyme that when elevated is associated with liver disease and liver damage. They compared GGT laboratory reference ranges considered normal for the baseline period, up to 51 U/L for men and 33 U/L for women, to individuals with higher GGT levels that they described as elevated. The same analysis was also performed for ALT levels. The prevalence of elevated ALT and GGT was found to be 13.5 percent and 13.2 percent, respectively. Mean GGT levels for those participants considered to have had normal baseline GGT measures were 20.1 U/L; and the mean GGT level of those considered to be elevated was 83.7 U/L. Ruhl and Everhart found that, whether in an age-adjusted-only model, or full multivariate-adjusted model, individuals with elevated ALT had approximately a fivefold increased risk of liver disease mortality. In contrast, liver disease mortality risks ranged between 13- and 19.4-fold for elevated GGT levels. The lower HR was attributed to the multivariate-adjusted model and the higher HR to the age-adjusted model. For mortalities attributed to diseases other than liver ones, elevated compared to normal GGT was reported to be associated with all-cause mortality in the age-adjusted model, HR 1.6; diabetes deaths, HR 4.9; as well as death from CVD and cancer, each having HRs 1.5; and other causes, HR 1.4. These HRs were generally consistent with those reported for the large Austrian Study (Kazemi-Shirazi et al. [15]) described above. The reason for the attenuation of HRs when comparing both studies, however, is mainly that this NHANES study compared the most highly elevated GGT levels as existing only among 13.2 percent of the entire study group to those of all the other participants having so-called “normal” GGT levels within a clinical laboratory range selected. The high HRs attributed the highest GGT category; 15 percent of the study population in the Austrian study was compared against a low-normal GGT range which included only 37 percent of the entire study population. In other words, in the NHANES study, the incremental GGT-related mortalities experienced within the central GGT percentiles in the US, or those between the 37th and 85th GGT percentiles, respectively, with increased mortality risks of 20%, 40%, and 67%, were subsumed in the reference group, thereby attenuating the HR difference when comparing this broader reference group to only those above the reference range ceiling.

Studies conducted in Europe have quantified the GGT-related risk of heart disease, cancer incidence, and cancer mortality among both men and women. Among these, Ruttmann et al. [18] reported cardiovascular disease mortalities among a cohort of 163,944 Austrian adults. CVD mortalities occurred in relation to GGT levels ranging from a 17% increased risk for men who had GGT measured in the range of 14–27 U/L, 28% increased risk for those with GGT of 18–24 U/L, 39% increased risk in the GGT range of 42–55 U/L, and a 64% increased mortality risk for those who had GGT levels that measured at 56 U/L or above; each of those risk categories was measured in comparison to those men who had GGT measured below 14 U/L (*n* = 29,320 or 39% of the male cohort). Similarly, fatal CVD events among women followed a dose-proportion relationship that became significant at GGT levels above 18 U/L with an incremental mortality risk of 35%; at GGT levels between 18 and 26 U/L, increased mortality risk was measured at 46%, and at levels above 36 U/L, it was shown to be 51%. However, compared to a majority of men (61%), CVD mortality risks were encountered by a much smaller (16%) minority of women. As noted above by Kazemi-Shirazi et al. [15] and the study reported from Finland [16], Ruttmann and colleagues preformed a subgroup analysis stratified by age and found stronger relationships between GGT and CVD mortality for both men and women younger than age 60 years than those who were older. Hazard ratios for incident CVD for younger men and women were 2.03 (1.53 to 2.69) for men and 2.60 (1.53 to 4.42) for women.

The connection to chronic kidney disease (CKD) is discussed more fully in Section 8. However, cardiovascular disease is a common cause of death among individuals suffering from CKD that often occurs in advanced stages of disease or End-Stage Renal Disease (ESRD). In 2008, Postorino et al. [19] tested the predictive power of GGT for cardiovascular and overall mortality in a cohort of 584 ESRD patients. During a four-year follow-up period, 194 patients died. Higher GGT levels predicted both all-cause and CVD mortality. The investigators noted “High GGT in ESRD patients is a strong, independent risk marker for all-cause and cardiovascular death. The predictive power of GGT for these outcomes likely reflects the involvement of this enzyme in oxidative stress mechanisms.” The investigators further noted that lower, rather than higher, body mass index (BMI) predicted mortality in ESRD. In support of their discussion of oxidative stress in uremia and its contribution to CVD, Postorino et al. cited a comprehensive review on the topic by Himmelfarb and colleagues [20], which incorporated by reference another informative review on catalytic metals, ascorbate and free radical damage authored by Buettner and Jurkiewicz [21]. Of course, a key metal most often described in connection to oxidative stress is iron.

In two additional studies from Austria, Strasak et al. [22, 23], quantified cancer incidence among cohorts of 79,279 men and 92,843 women who were followed up for a mean of 12.5 and 13.5 years, respectively. In their study of men, Strasak and colleagues observed that incident cancers occurred in dose-proportion to baseline GGT levels (*P* for GGT log-unit increase < 0.0001;* P* for trend < 0.0001). They further noted that GGT was significantly associated with malignant neoplasms of digestive organs, the respiratory system/intrathoracic organs, and urinary organs (all* P* < 0.0001). As noted above by Ruttmann et al. for CVD, age of participants significantly modified the association of GGT and cancer risk (*P* < 0.001). Cancer risk was noted to be markedly stronger in participants aged less than 65 years. Strasak et al. found similar results for incident cancers in their female cohort where 4,884 cancers were observed, although the age effect noted for men was not observed among women. Compared to normal low GGT (<17.99 U/L), cancer risk was elevated for all other GGT categories (*P* for trend < 0.0001), with adjusted hazard ratios of 1.06 (0.99–1.13) for GGT levels between 18.00 and 35.99 U/L, 1.12 (1.02–1.22) for levels between 36.00 and 71.99 U/L, and 1.43 (1.28–1.61) for GGT (>72.00 U/L). In the women's study cohort, GGT significantly increased the risk for malignant neoplasms of the respiratory system/intrathoracic organs, digestive organs, breast and female genital organs, and lymphoid and haematopoietic cancers (all,* P* < 0.006).

A recent study from Korea monitored a population of 1,662,087 Koreans over a 17-year period and found a positive gradient of HR for cancer in relation to GGT quintiles. The highest quintile had 6-7-fold higher risk to liver cancer, as well as significant increased risk for cancers of the esophagus, larynx, stomach, colorectal, bile duct, and lung [24]. Note that the multiple fold increases described here for liver cancer are similar to those reported above by Kazemi-Shirazi et al. in the large study from Austria. In 1974, Lum [25], a New York physician, reported that very high serum GGT levels, multiple times the upper normal GGT range limit, were observed among patients with biopsy-proven, clinically evident metastatic carcinoma of the liver and also patients with carcinoma of the pancreas, as well as with carcinoma of the gall bladder.

Thus, in summary, large population-based studies from Austria, Finland, Korea, and the United States all show a significant increased disease and mortality risk linked to elevated GGT levels, with statistically significant links to MetS, CVD, heart failure, cancer, stroke, diabetes, and all-cause mortality.

## 3. GGT: In Vascular Impairment and Antioxidant Depletion, and Related Disease Mechanisms

The inflammation observed with most diseases of aging is strongly associated with C-reactive protein (CRP) and high-sensitivity, or hs-CRP measures, both having proven utility as markers of inflammation. In 2004, Lim et al. [26] established the significant relationships between GGT levels and oxidative stress by demonstrating in the NHANES III population that, as serum GGT increased through all deciles, its movement maintained an inverse relationship to six of seven serum antioxidants, all with *P* < 0.01; the one exception was vitamin E, *P* = 0.22. These associations were further confirmed in 2004 by Lee et al. [27] in the Cardiovascular Risk Development in Young Adults (CARDIA) Study. The next year, Lee and Jacobs Jr. [28] demonstrated that “the strong association of serum GGT and CRP” had existed for each gender and among all ethnic categories. Their conclusions were also based on an analysis of NHANES III data. In 2011, a study reported by Kim et al. [29] found that earlier reported associations from the US and other Western countries could be verified in a large study of 42,906 Koreans aged 20–86 years. Among 30,710 study participants who had complete data, the 10-year Framingham Risk Scores correlated significantly with GGT levels throughout each GGT quartile after adjustment for age, gender, alcohol consumption, and all other known coronary artery disease risk factors. In a 2013 report from Turkey, Atar et al. [30] found increasing prevalence of coronary artery calcification (CAC) in individuals within the higher GGT quartiles. A similar association between GGT and CAC was found in Korea among 14,439 males and females without coronary artery disease. Lee et al. [31] found a dose-response relationship between GGT quartiles and CAC prevalence. The prevalence of CAC was 4.6 percent among participants in the first GGT quartile, 8.7 percent in the second, 11.8 percent in the third, and 14.7 percent in the fourth GGT quartile. In another study undertaken in Turkey, Celik et al. [32] compared 138 male and female subjects with coronary plaques to 121 without such plaques. Subjects with plaques had mean GGT levels of 35.7 U/L, while the levels of those without coronary plaques were 19.6 U/L. Other significant correlates of GGT and coronary plaques were smoking, diabetes, hypertension, hyperlipidemia, creatine, triglycerides, uric acid, HbA1c, and hs-CRP. Cross-sectional study data such as these show the relationship of these common CVD risk factors with elevated GGT levels.

Lee and Jacobs, authors or coauthors on several articles and studies discussed in this review, presented their study findings of elevated GGT in association with increased levels of urinary metabolites from xenobiotics with short half-lives. Their 2009-reported study [33] was based on data obtained from 1,256 adult participants in NHANES 2003-2004. Lee and Jacobs determined the association of serum GGT with 10 monohydroxy-polycyclic aromatic hydrocarbons (OH-PAHs), which are pollutants formed by incomplete combustion of organic materials. Among the 10 OH-PAHs they investigated, significant positive correlation with serum GGT levels was reported for eight of them. They proposed that GGT is needed to break down the glutathionylated xenobiotics that are formed by the liver as a step towards detoxifying these molecules. Lee et al. [34] had earlier reviewed reports associating moderately elevated GGT levels in connection to xenobiotics having longer half-lives, including heavy metals, dioxins, and organochlorine pesticides. Their review described the association of GGT levels to what they referred to as persistent organic pollutants (POPs). They suggested that the established relations between GGT levels and T2D might be related to xenobiotics found in the environment. They provided an elegant description of the necessity of GGT for the effective detoxification and elimination of these xenobiotics. Lee et al. also discussed how this process might lead to the depletion of antioxidant levels, increased oxidative stress, and the onset of such conditions as T2D.

As noted in Introduction, and as observed by Aberkane et al. [6], damaged red blood cell (RBC) membranes will cause the leakage of potentially toxic iron into the serum. This leakage can range in degrees from acute to chronic, as well as the associated damage that it can cause. Recently, several studies of bottlenose dolphins conducted by the US Navy and others have demonstrated this effect. Venn-Watson et al. [35] noted in their study on MetS in bottlenose dolphins (*Tursiops truncatus*) that MetS, which is common to more than one in every three adults in the US, is associated with high serum ferritin (SF) levels and iron overload in both humans and dolphins. Captive dolphins are particularly susceptible to MetS because their diets are normally constrained by a human selection process. This study showed that when subject dolphins affected by MetS were provided diets with increased intake of hepatadecanoic acid (C17:0), a saturated fatty acid, their MetS, was resolved along with significant reductions in iron levels as measured by SF, accompanied with normalization of glucose, triglyceride levels, and insulin. The investigators described the dietary change that produced this result as comparable to providing butter in exchange for margarine containing plant-based trans-fatty acids. They noted that “butter had 10-fold higher levels of C17:0 compared to the next higher level of C17:0 foods.” The mechanism suggested as responsible for these metabolic improvements was a significant strengthening of RBC membrane integrity, which resulted from the addition of these saturated fats to the diet. Venn-Watson stated in a recent interview [36], “We saw blood ferritin levels decrease in all six dolphins within three weeks on their new diet.” Interestingly, previous investigations of iron overload in dolphins have been centered on the traditional use of therapeutic phlebotomy to reduce toxic iron stores. In a 2009-reported study by Johnson et al. [37], phlebotomy treatment was instituted to reduce iron levels of three Atlantic bottlenose dolphins that had been diagnosed with poor appetite, malaise, significantly elevated liver enzyme activities (AST, ALT, and GGT), high serum iron concentrations, and elevated serum iron saturation (>80%). During a two-year treatment period, weekly phlebotomy resolved the markedly high pretreatment serum liver enzyme activities that, according to Johnson et al., “were also indicative of excessive iron deposition and cellular damage in other tissues as well.” Two of the animals had pretreatment serum GGT levels that were between 8- and 18-fold times the upper end of the dolphin GGT reference range. Phlebotomy treatments successfully lowered GGT levels to within the animal's normal range; these treatments similarly reduced their pretreatment levels of serum iron, AST, and ALT to within normal reference ranges as well. Phlebotomy treatment to reduce excess iron stores in humans has been proven as effective therapy for conditions of hereditary and acquired iron overload; recently it has emerged as an effective treatment in several metabolic conditions, including fatty liver disease (NAFLD) with hyperferritinemia. This was demonstrated by Valenti et al. [38] in a 2014-reported, controlled clinical trial of iron reduction in NAFLD patients. As observed in the above Venn-Watson et al. and Johnson et al. dolphin studies, significant metabolic improvements, including reduction of liver enzymes, inclusive of GGT, were achieved in the phlebotomy treatment arm of the Valenti et al. trial.

Venn-Watson supported their novel finding and demonstrated its potential for beneficial translation to human populations by citing and discussing several relevant references. We reviewed two of these references here: the first is a 2015-published review by Jenkins et al. [39] that discusses consumption of pentadecanoic acid (C15:0) and C17:0 in connection to human health and disease. The authors cite literature in which reduction of metabolic and psychological disease incidence, improved disease prognosis, and inverse relationship to disease development have been associated with increased consumption of odd chain fatty acids. We also reviewed a second Venn-Watson et al. cited study, this one by Pereira et al. [40], who analyzed dairy consumption frequency data provided by 3,157 young American Black and White adults who participated in the CARDIA study. This investigation found a dose-proportional relationship between increased consumption of dairy products and reduced incidence of MetS (insulin resistance syndrome) and also for several MetS component conditions, including obesity, hypertension, and abnormal glucose levels. However, these findings were only realized as significant among participants who were overweight or obese.

Chemical iron chelation treatment has been shown to maintain normal GGT levels in people affected by potentially severe iron overload caused by multiple blood transfusions. *β* thalassemia (*β*-thal) is one important example of this, as it is a genetic condition that cannot be treated by therapeutic phlebotomy. In 2011, Abdalla et al. [41] reported the results of a study of 40 Jordanian *β*-thal children (patients) and 40 controls, all under the age of 13 years. The investigators found that serum GGT levels measured in both patients and controls were statistically the same, respectively, 11 and 10 U/L. Since all patients had been receiving iron chelation therapy, the investigators noted that this was an indication of effective treatment, meaning the chelation treatment had effectively reduced loosely bound, potentially toxic, free iron in the serum to the same levels as those of the unaffected children. Although the patients in this study still had significantly higher iron stores, as measured by SF, the chelation therapy had apparently worked. This might be a strong indication that serum GGT is a valid marker of potentially toxic free iron, which is often referred to as non-transferrin-bound iron (NTBI). The investigators summarized their findings as follows:

“Our study suggests that in *β*-thal, the first target organ dysfunction is the liver, enhanced by an iron overload leading to a status of oxidative stress and initiates free radical reactions. Proper iron chelation and administration of adequate antioxidants may represent a promising way of counteracting oxidative stress and its deleterious effect on the prognosis of the disease.”

In Section 2, we discussed serum GGT-related increased all-cause mortalities, incident MetS, and heart failure that were experienced by participants in the Framingham Offspring Study (FOS). The studies by Lee et al. [9] and Dhingra et al. [10] both showed that morbidity and mortality were approximately 2-fold higher among participants having baseline GGT levels (as measured between 1978 and 1982) above their respective gender-related median level of 9 U/L for women and 16 U/L for men. Approximately 20 years after FOS baseline GGT measures were obtained, serum GGT and other blood values, including *α*- and *β*-carotene, lycopene, and vitamin E concentrations, were measured in a study of 185 Dutch male and female volunteers (age 35–64) who were described as healthy. Alarmingly, baseline GGT levels among these participants were 70% higher than they were among the FOS participants measured just 2 decades earlier. The objective of this study [42] was to test the efficacy and safety of a plant sterol ester-enriched food spread and to measure anticipated reductions in total and LDL-cholesterol levels. All of the subjects were qualified as regularly using plant-based spreads before study entry, after which they were randomized to two cohorts, a control cohort provided a normal spread and a subject cohort provided plant sterol-enriched spread that contained 1.6 g of free plant sterol equivalent per day. The study was conducted over a 52-week period. By the end of the study period, and compared to controls, subjects who received the sterol ester-enriched spread experienced an average 4% reduction of total cholesterol and 6% reduction of LDL-cholesterol. However, importantly, GGT levels, already much higher than those of the FOS participants at the beginning of this study, increased more than 9% in the control cohort and 16% in subject cohort members, respectively, during this short, one-year study; and concurrent with increases in GGT, AST and ALT levels increased in subjects, and levels of *α*- and *β*-carotene concentrations decreased significantly in subjects (*P* < 0.001). The inverse, graded relationship between GGT and carotenoids, and several other antioxidant vitamins, was noted by Lee et al. [43] in 2006 based on NHANES participants.

In contrast to the slower leakage of RBC contents that occurs with conditions such as MetS, acute rupture of RBC membranes can quickly lead to severe clinical situations. The recent outbreak of Ebola hemorrhagic fever that occurred in West Africa provides an example of such abrupt RBC leakage. Investigators from the US Centers for Disease Control and Prevention (CDC) and Emory University School of Medicine reviewed serum samples that had been stored since a large earlier outbreak that occurred in Uganda in 2000 and 2001 [44]. The investigators noted that hemorrhage, which is certainly a hallmark of Ebola* hemorrhagic* fever, had been shown to occur in something less than half the cases of Ebola infection. A potentially key observation from this study was that, although hemorrhage alone did not always correlate with fatality, serum ferritin levels did and did so during each stage of infection. Based on both the extremely high and relatively high SF measures reported by the investigators, it appears that massive releases of RBC iron occurred in fatal cases to a much greater degree than in the nonfatal cases. Further quantification of the association of high GGT and fatal Ebola can be gathered from a primate euthanasia study [45] conducted by the US Army in an investigation of the experimental infection of rhesus monkeys (*Macaca mulatta*) with the Ebola virus. Serum GGT proved to be one of the key factors differentiating surviving monkeys from those that succumbed to the infection. In this study, elevations of GGT were significantly related to a negative survival outcome (OR = 17.1 (1.03, 269);* P* < 0.05).

Curcumin, an Indian spice found in turmeric and curry powder, has been shown to ameliorate many diseases linked to elevated GGT. Perhaps the most significant protective feature of curcumin is its ability to chelate free iron [46]. In a toxicology study comparing the effects of curcumin and an analogue of curcumin in carbon tetrachloride-induced hepatotoxicity in rats, Kamalakkannan et al. [47] used six groups of six rats each to test whether, and to what degree, curcumin or a curcumin analogue (BDMC-A) would reduce markers of oxidative stress and improve levels of antioxidant defenses to the greatest degree. Among three groups of carbon tetrachloride (CCl_4_) treated rats, both curcumin and its analog significantly helped preserve glutathione, vitamin C, and vitamin E levels compared to rats that were not administered either of the curcumin oral preparations. Although both preparations were significantly effective, between curcumin and BDMC-A, the protective effects of BMDC-A were superior to those of curcumin alone (*P* < 0.05). Kamalakkannan et al. noted that “the trichloromethyl radical to cell protein is considered the initial step in a chain of events that eventually leads to lipid peroxidation of the cell membrane and endoplasmic reticulum.”

Consistent with the discussions in this section, in 2002, Baillie-Hamilton [48] hypothesized that long-term exposure to low doses of chemical toxins promoted weight gain in humans by altering endocrine function and body weight set points. We discussed earlier that elevated GGT is an* early* disease marker. We will further confirm that below, with recently established findings indicating that elevated GGT, as well as SF, precede the transition to obesity and MetS, at least in the populations reported below. We will clearly demonstrate this more completely in the following section, describing a series of longitudinal studies reported by investigators in Korea.

## 4. GGT: A Predictor of Obesity, MetS, Prehypertension, and Insulin Resistance

As noted above and earlier based on baseline GGT levels measured in the Framingham Offspring Study (FOS), only moderately elevated GGT, starting at levels just above population GGT medians, and often measured one or two decades in advance of disease end points or mortalities, established GGT as an early predictive marker of MetS, CVD, heart failure, and all-cause mortality. Several more recent and larger longitudinal studies undertaken in Korea have also demonstrated that moderately elevated GGT predicted the incidence of MetS and other commonly accepted disease risk factors, including obesity, prehypertension, and insulin resistance. In Section 3, we discussed the potential association of environmental toxins with T2D. Obesity is a medical condition often associated with increased risk for T2D. However, in a 2013 study of 18,510 nonobese Korean men (mean age of 43 years), Suh et al. [49] reported that, during a mean 3.62-year follow-up, 14.6 percent of the men became obese. All 18,150 study participants had been within the normal BMI category (body mass index under 25) at baseline. The hazard ratios (HRs) for progression to obesity, when all quartiles were compared to subjects in the first GGT quartile, were 1.23 for men in the second GGT quartile and 1.35 and 1.54, respectively, for men in the third quartile and fourth GGT quartiles. Progression to severe obesity (BMI ≥ 30) was significant for third and fourth quartile GGT levels, as indicated by HRs of 2.05 and 2.52, respectively. This progressive risk relationship was significant as well for increases in waist circumferences to greater than 90 centimeters for men in each GGT quartile above the first, HRs, 1.56, 1.59, and 2.00, for quartiles two through four.

In an interesting study from Bangladesh, a country not known for obesity, Alam et al. [50] reported that, among 493 patients, 177 were evaluated and confirmed with a diagnosis of either nonalcoholic steatohepatitis (NASH) or fatty liver disease (NAFLD). After eliminating the potential influences of conditions often associated with serious liver disease, including hepatitis B and hepatitis C, possible adverse reactions to certain pharmaceutical drugs, autoimmune liver disease, and conditions such as hemochromatosis or Wilson's disease, conditions characterized by iron and copper overload, respectively, the investigators determined that only serum GGT levels and presence of diabetes differentiated the NAFLD patients (*n* = 102; GGT 40.2 U/L) from those with NASH (*n* = 75; GGT 51.7 U/L, *P* = 0.05). There were no other anthropometric or biochemical differences noted between the two subject groups. Alam et al. noted reports that increased GGT levels had been reported as a risk factor for advanced fibrosis and that the insulin resistance associated with diabetes and other conditions can worsen diabetes and contribute to a vicious cycle. We will demonstrate both directly below and further below in Section 5 how iron metabolism is synergistically related to these dysmetabolic features.

The blood marker considered most appropriate for measuring body iron storage is serum ferritin (SF). In another study from Korea reported in 2014, Park et al. [51] reviewed the baseline serum ferritin (SF) measures of 17,812 normal BMI Korean men who were followed up for 3.45 years. Similar to the Suh et al. GGT findings for obesity, SF levels, beginning in the third SF quartile (SF above 96.9 *μ*g/L) indicated significant risk of progression to obesity, severe obesity, and increased waist circumferences in dose-proportional relationships. Although the HRs were somewhat lower for SF than for GGT, both measures were independently predictive of obesity onset and severity, and all *P* trends for both measures were <0.001 for each end point studied. Using similarly structured studies as they had for obesity, Park et al. [52] and Suh [53] also determined that GGT and SF were significantly predictive of incident MetS. Park and colleagues demonstrated that SF above 61.5 *μ*m/L, the second quintile SF entry point, was independently predictive of incident MetS during 3.51 years of follow-up.

By the end of the follow-up period, 2,177 men (11.4 percent of all participants) had developed MetS. The HRs (compared to the first quintile) for incident MetS ranged from 1.26 to 1.70 for quintiles two through five; and all obtained *P* < 0.001. Park et al. also reported that the intraquintile mean GGT levels (also measured in this study) for each SF quintile one through five, respectively, were 23 (16–35), 26 (18–39), 27 (29–41), 30 (20–46), and 35 (23–58). Suh reported similar results for GGT, as Park et al. did for SF, for forecasting MetS based on baseline GGT quartiles. As noted for obesity, the GGT HRs were greater than for SF. They ranged from <17 U/L for the upper end of the first (or referent) quartile, to HRs of 1.57, 2.73, to 3.78, respectively, for the second, third, and fourth GGT quartiles. Park et al. also calculated the relationship of SF to GGT in each of their SF quintiles, and for each of three risk models. Overall, these biomarker relationships were consistent with results reported by Targher et al. [54] in 2007 in their published comment to a review entitled “The Role of Iron in Diabetes and Its Complications,” authored by Swaminathan et al. [55]. Targher and colleagues reported findings from their study of 2,449 individuals referred for blood tests in which both SF and GGT had been measured. Although SF levels alone were associated with both impaired fasting glucose (IFG) and incident diabetes, when evaluating the relationship of SF and GGT, they discovered the following interactions (as described in the excerpt below):

“Although the prevalence rates of ferritin quartiles increased steadily across IFG/diabetes categories (ranging from 17 to 27% for IFG and from 4 to 8% for diabetes; *P* < 0.0001), these prevalences remarkably varied by GGT quartiles. As GGT increased, the prevalence rates of ferritin quartiles across IFG/diabetes categories strengthened (*P* < 0.001 for interaction). For example, within the lowest GGT quartile, ferritin quartiles were not associated with IFG (ranging from 12.7 to 14.5%) or diabetes (from 1.2 to 1.5%), in contrast to the highest GGT quartile, wherein the prevalence rates ranged from 19.2 to 28.3% for IFG and from 9.4 to 13.5% for diabetes (*P* < 0.01). These results remained significant even after adjustment for sex, age, lipids, and hs-CRP.”

Since diabetes (mainly T2D) and impaired fasting glucose are the most common adverse conditions associated with MetS, the strong interaction observed by Targher et al. might significantly enhance prognostic capabilities of a simple blood test in predicting MetS, IFG, T2D, and atherosclerosis (the second most common condition predicted by the development of MetS).

Very recently, Wei et al. [56] similarly reported highly relevant findings that also support the association of elevated SF and GGT in individuals found to have MetS. In their study of 1,024 individuals, referred to as the Chinese Yi ethnic group, Wei et al. found that both SF and GGT were significantly higher in those with MetS when compared to those without MetS. Serum GGT was correlated positively with SF (*P* < 0.05). Interestingly, although most of the studies summarized in this section surveyed disease risks in male populations, this study, as well as the liver disease study from Bangladesh [25], found that high GGT and SF, even when found in* women* at levels below those of men (GGT 32.0 U/L versus 63.0 U/L) and for SF (SF 117.7 *μ*/L versus 272.9 *μ*/L), were related to a higher MetS prevalence among women than men. Those disparate findings relative to gender differences were more pronounced when the criteria devised by NCEP-ATP III [57] were used than when compared to criteria proposed by the China Diabetes Society (in Chinese). The NCEP-ATP III model is the model commonly used in the US and in many Western countries. The researchers described “*The Association between GGT and Ferritin*” as follows:

“Spearman correlation analysis showed that GGT was significantly correlated with ferritin (*r* = 0.425, *P* = 0.000), and both of them were significantly correlated with gender (*r* = −0.499, *P* = 0.000 for ferritin; *r* = −0.277, *P* = 0.000 for GGT). Spearman correlation analysis confirmed that GGT was significantly correlated with ferritin in both genders (*r* = 0.256, *P* = 0.000 for females; *r* = 0.437, *P* = 0.000 for males).”

Importantly, Wei et al. reported that “the increased risk of having each of the metabolic syndrome components (overweight or obesity, hypertriglyceridemia, hypertension, hyperglycemia, and insulin resistance) was also observed in those subjects after adjustment for possible confounders (*P* < 0.05).” Wei and colleagues suggested as their concluding point in their abstract: “These data indicate that GGT and ferritin synergistically correlate with the risk of the metabolic syndrome, suggesting that they could potentially be used as predictive biomarkers for the metabolic syndrome.”

Other research reported in this section and elsewhere in this review demonstrates that several other MetS component factors are independently associated with serum GGT pursuant to dose-dependent relationships. Two such other predictive components of MetS include prehypertension (as a precursor to hypertension) and insulin resistance; the latter pertains to some MetS definitions only. Incident prehypertension was studied by Chun et al. [58] in a cohort of 13,345 healthy Korean men (mean age 42.9 years) who were followed up for a median of 2.8 years (45,919 person years). During the follow-up, 7,867 men (58.6 percent) developed prehypertension. The hazard ratio model was adjusted for insulin resistance, triglyceride, HDL-cholesterol, total cholesterol, log hs-CRP, and creatinine. The risks of developing prehypertension followed a dose-response relationship for all GGT levels above the first GGT quartile (GGT less than 17 U/L). For GGT quartiles two through four, the HRs (all significant within 95% CIs) for incident prehypertension were 1.19, 1.18, and 1.35, respectively, *P* < 0.001. In another study, the development of insulin resistance, considered a key risk factor for MetS, T2D, and CVD, was reported by Ryoo et al. [59] in 2014. The latter report described the relationship of baseline GGT to the future development of insulin resistance in a cohort of 22,931 Korean men followed up for 3.54 years. The results were very similar to those reported in the other above studies from Korea. GGT levels above the first quartile, measured at study entry, indicated HRs for developing insulin resistance of 1.19, 1.38, and 1.58 for quartiles two through four, respectively. All HRs were significant within a 95% CIs, *P* < 0.001. Over the study period, 3,856 men developed insulin resistance in a dose-dependent fashion across each of the second, third, and fourth GGT quartiles, compared to the first GGT quartile.

## 5. GGT: A Predictor of T2D and Gestational Diabetes 

In the study described directly above, GGT levels relative to the future development of insulin resistance is of special interest because insulin resistance has recently been noted to be a key factor, when combined with serum GGT levels, for forecasting future development of gestational diabetes (GDM). In 2014, Sridhar et al. [60] reported findings from their case-control study of a large cohort of young women (*n* = 4,098) in Northern California who were tested between 1984 and 1996 for levels of both insulin resistance and GGT. Testing was on average completed seven years prior to either the woman's first or next subsequent pregnancy. A total of 267 women experienced GDM during their subsequent pregnancy. Among the women with BMI levels above 25, those with GGT levels above the cohort median of 18 U/L experienced GDM at two times the rate of women in the first GGT quartile (GGT 8.0–13.4 U/L). While women with BMI over 25 at study entry in the second GGT quartile (GGT 13.5–18.0 U/L) did not experience increased GDM risk, women in the second GGT quartile having BMI* below* 25 at study entry experienced a 1.8-fold increased risk of GDM. The risk of lean women was similar to that of the heavier women, when GGT levels were above the median of 18 U/L. Notably, Sridhar et al. reported that, when the interaction of insulin resistance (assessed by HOMA-IR) was added to the risk analyses, the HRs changed markedly. Women who had GGT above the first quartile (13.5 U/L), together with insulin resistance measured in the upper tertile of HOMA-IR, experienced increased GDM HRs, compared to first quartile GGT and HOMA-IR (in the lower two tertiles), of 2.7, 3.8, and 4.9 in the second, third, and fourth quartile of GGT, respectively. Interestingly, GGT alone, as reported in any quartile in this study, did not pose a risk of GDM when insulin resistance (assessed by HOMA-IR) was measured in the lowest two HOMA-IR tertiles. The significant interaction noted here was very similar to that noted above by Targher et al. [54] when serum ferritin and GGT measures were analyzed to both assess the risk of incident IFG and diabetes in their study cohort and calculate the interaction of each of these risk markers. Thus, GGT interacts with both serum ferritin and insulin resistance to enhance disease risk.

Many investigators have explored and described the role of iron in GDM. Recently Zhuang et al. [61] reviewed 32 articles cited in MEDLINE/PubMed that were among 85 publications yielded in a search using appropriate key words. Excess iron ingestion (primarily heme iron) or total iron, as well as high or high normal iron stores measured by SF, all prior to and/or during pregnancy, has been broadly identified as risk factors for GDM. Generally, the mechanisms discussed and described relating to GDM included the elevation of oxidative stress levels leading to lipid peroxidation, DNA and cell damage, and observations of residual serum or urine markers that indicated oxidative stress. Notably, GGT itself has been shown to be a valid marker of oxidative stress, and iron is a well-established prooxidant. We believe that, in 2004, Lee et al. [62] were the first to review dietary heme iron in direct relation to increased oxidative stress markers and GGT, together with the concomitant depletion of important antioxidant vitamins and glutathione. Their data and information had been obtained from epidemiological studies conducted mainly in the US. Very recently, Zein et al. [63] noted that the risk of GDM might be increased in women with increased iron stores in early pregnancy as measured by SF. They conducted a prospective, observational study involving 104 nonanemic women. They divided the cohort into four groups during the first trimester of pregnancy based on SF quartiles. All participants were screened for GDM with 75-gram oral glucose tolerance tests (OGTT) at 24–28-week gestation. The investigation determined that early pregnancy SF levels were significantly correlated with glucose levels after challenged by one- or two-hour OGTT, and that SF greater than 38.8 *μ*g/L, as was found in women having fourth quartile SF, resulted in significantly higher GDM risk.

Many investigators have associated moderately elevated SF with GDM. For example, in 2014, Javadian et al. [64] in Iran, using a case-control study design, compared a group of 52 pregnant women with GDM to 50 normoglycemic pregnant women. Although both groups were demographically similar, women with GDM had higher SF (31.2 *μ*g/L versus 24.8 *μ*g/L), *P* = 0.012; hemoglobin (12.9 g/dL versus 12.2 g/dL) *P* = 0.005; and malondialdehyde (MDA), which results from polyunsaturated fatty acid degradation in the presence of excessive reactive oxygen species (ROS) generation (1.22 mM versus 0.57 mM), *P* = 0.0001. Although not measured by Javadian et al., the existence of increased oxidative stress as measured by MDA serves as an excellent marker for increased GGT, as was the case in the above-described Sridhar et al. study from Northern California. In another study from Iran, Amiri et al. [65] similarly measured SF levels and found a direct relationship in pregnant women with GDM when compared to those without (52.1 *μ*g/L versus 30.4 *μ*g/L), *P* = 0.0001. As in the study directly above and the study conducted by Zein et al., OGTT was used to determine GDM cases. Amiri et al. further noted that SF above 80 *μ*g/L increased the risk of GDM 2.4-fold.

Similar to serum GGT, serum ferritin levels (SF) can be easily measured by a simple blood test. Even among healthy young people, SF and iron stores can be reduced by blood donation or therapeutic phlebotomy. Phlebotomy has been demonstrated to reduce SF, and multiple other disease markers, including GGT, in patients with chronic hepatitis C [66]. In the case of pregnant women, excessive iron stores during pregnancy can be moderated by reducing or ceasing iron supplementation when iron stores are adequate, or by making appropriate dietary adjustments, including lowering dietary iron intake. When conditions of disease risk, such as prediabetes, insulin resistance, or prehypertension, exist before pregnancy, supplementation of alternative nutrients such as curcumin extract might be considered. Curcumin has been noted to enhance several protective functions, including improving glutathione status, by lowering serum GGT levels [67] and by chelating potentially toxic iron as described by Minear et al. [68]. In a recently reported randomized, double-blinded, placebo controlled trial, undertaken in Thailand, subjects randomized to the treatment arm received curcumin (the main curcuminoid found in turmeric) in extract form. Chuengsamarn et al. [69] reported that, among biomarker improvements achieved by subjects in the treatment versus placebo arms, perhaps the most notable was reductions in levels or insulin resistance (as measured by HOMA-IR). Subjects who received curcumin extract achieved the following (mean) reductions in HOMA-IR levels versus the levels of subjects in the placebo control group: three months 0.37 (not significant), six months 0.64 (*P* < 0.05), and nine months 0.86 (*P* < 0.001). The curcumin arm subjects also achieved improvements in body weight (lowering) and waist measurement, both* P* < 0.05 over nine months, fasting plasma glucose* P* < 0.01 at three, six, and nine months, two-hour OGTT* P* < 0.01 at each time interval, and lower HbA1c also* P* < 0.01, also at each time interval. Several other significant improvements were also noted. Importantly, the investigators found the treatment to be safe, and no subjects in the curcumin treatment arm converted to a diagnosis of diabetes, while 16.7 percent of the subjects in the placebo arm were diagnosed with diabetes by the end of the one-year study.

## 6. GGT: Mortality Perspectives from the Life Insurance Industry

Medical science researchers have described the utility of GGT as a predictor of all-cause mortality only during the last fifteen years. However, GGT data has been collected by the life insurance industry for many years. Their principal reason for measuring GGT levels was its ability to mark alcohol abuse or risky drinking behavior in life insurance applicants. When high GGT levels were found in life insurance candidates, premium policy pricing or even application rejection often resulted. In 2012, Palmier et al. [70] published their analysis of life insurance industry data entitled “Leading Contributors to Mortality Risk in Life Insurance Applicants.” They noted that “stratification of mortality risk was a central function of (life insurance) actuaries.” Palmier et al. investigated mortality statistics based on deaths reported from a portfolio of more than 6 million life insurance applicants. They grouped applicant data into five underwriting brackets, one of which was GGT combined with alkaline phosphate (ALP). For male applicants, and in each of four age categories, GGT-ALP ranked highest for predicting mortality risks when compared to four other major mortality risk categories. This ranking applied for all men in age groups 18 through 59 years. For men aged 60 through 79 years, GGT-ALP ranked second behind aspartate aminotransferase and alanine aminotransferase (AST/ALT). For women, GGT-ALP was ranked first for those aged 30 through 59, and second behind urine protein and urine creatinine for ages between 60 and 79 years. Interestingly, with one exception, among young women aged 18 through 29, a lipid panel ranked either third or not at all, through all five female disease risk categories, and ranked only as high as fourth or fifth in three of five male age categories. Palmier et al. noted as part of their closing of the “emerging consensus” that liver function tests, “*particularly GGT*” had become central to the life insurance underwriting process.

Palmier and Lanzrath published a second study in 2012 that was specific to determinants of cancer mortalities [71]. They reviewed complete laboratory and physical measurement profiles of 1.250 million life insurance applicants that were followed up for 4.7 years for insurance claims resulting from 518 cancer deaths. They found that “among serum and urine analytes, liver function tests (principally GGT and ALP), hypocholesterolemia, proteinuria, and low fructosamine were found to be independently predictive of cancer mortality.” In their Figure 1, Palmier and Lanzrath provided a hazard ratio (HR) curve for males 40 through 49 years of age that depicted an increasing HR trend starting at about 0.70 to 0.80 for GGT between approximately 15 U/L and 20 U/L that became a fairly flat curve between GGT levels of approximately 45 U/L to 85 U/L. The mortality HRs at these higher GGT levels ranged between approximately 1.4 and 1.5. This approximate doubling of HRs between low to high GGT levels was consistent with the HRs reported for cancer mortalities in the large Austrian population study reported above [15] by Kazemi-Shirazi et al. Interestingly, Palmier and Lanzrath reported that low, rather than high, cholesterol was independently predictive of cancer mortality and, that there was no identifiable relationship between BMI levels and cancer mortality; an observation they noted was at variance with one similarly large prospective cancer mortality study reported in the US in 2003 by Calle et al. [72]. The Palmier and Lanzrath finding respecting body weight and cancer was recently replicated in a study of 175 cancer patients assessed before chemotherapy by Gonzalez et al. [73]. The apparent differences in whether or not obesity is or was a factor in cancer mortalities, when comparing findings from the life insurance industry and those described by Calle and colleagues, might be a factor in what has recently become known as the “obesity paradox.” The Calle et al. study was published in 2003, during a time when the obesity paradox was just starting to be reported by researchers. Since 2003, there have been more than 750 studies reported generally consistent with this report by Palmier and Lanzrath suggesting that, for a growing number of potentially fatal disease conditions, obesity, even if it encourages disease initiation, might protect against fatal consequences. This survival paradox was recently dramatically demonstrated in a meta-analysis of nearly 3 million pneumonia cases by Nie et al. [74].

## 7. GGT: Ethnic Differences in Oxidative Stress and Iron Measures

The NHANES III study described above provided fertile grounds for researchers to investigate multiple biochemical markers relative to gender, ethnic, and socioeconomic differences that might form the basis for disparate disease incidence rates and health outcomes among ethnically diverse US resident groups. The NHANES III population, by design, contained disproportionately greater numbers of African American men and women than their actual percentages in the US population. NHANES researchers referred to this cohort as non-Hispanic Black (NHB). In 2008, Pan and Jackson [75] studied ethnic differences between NHB men and White male participants. The latter group was referred to in this NHANES study as non-Hispanic White (NHW). Pan and Jackson reviewed data from 3,554 subjects, aged 20–65 years that included 1,938 NHW and 1,626 NHB men. Among other measures evaluated, Pan and Jackson analyzed differences in levels of the iron storage protein, serum ferritin (SF), and serum GGT.

In 2000, Zacharski et al. [76] had noted the significant differences among ethnicities when comparing serum ferritin (SF) and body iron stores among adult NHANES III participants (a total of 20,040 individuals over the age of 17 years). They described and charted those differences as noted further below. In this study, Zacharski et al. established that NHB men had significantly higher iron stores (as measured by SF) than NHW men. They also noted that NHB men and NHB women experienced an approximately 1.5-fold increased mortality rate compared to NHW men and women and that NHBs were disproportionately affected by chronic diseases including higher stroke, heart attack, cancer, and diabetes rates. Zacharski et al. suggested that the significantly higher SF, as noted particularly in the NHB men, might provide support for the controversial hypothesis put forth by Dr. Sullivan in 1981 and 1992 [77, 78]. Sullivan was first to hypothesize that the comparatively low SF levels, normally measured in menstruating women, that resulted mainly from iron lost through menstruation, childbirths, and lactation, translated to a survival benefit for younger women. NHANES III data helped demonstrate dramatic gender differences in SF levels. SF is generally described as the most accurate blood marker of body iron stores. Gender differences were demonstrated in a graphic (Figure 2) in the Zacharski et al. study. Figure 2 also demonstrates significant ethnic differences between NHB men (from age 17 years) and NHB women (from about age 45), when both groups are compared to their NHW age-matched counterparts.

Unfortunately, Zacharski et al. did not compile GGT data from NHANES III. Understandably, few of the epidemiologic and other research data (such as described above) had yet been developed or published. That only happened after 2000, the year this Zacharski et al. study was published. However in 2008, both serum ferritin and GGT data from NHANES III were compiled by Pan and Jackson, as noted above. Among several different categories of serum data from NHANES III that Pan and Jackson analyzed, only serum GGT was found to be positively associated with SF. A similar significant GGT levels difference (*P* < 0.0001) was observed between young Black and White adults by Patel et al. [79], based on data from the Bogalusa Heart Study. And, as had been noted earlier from Zacharski's NHANES III data, SF measures observed by Pan and Jackson for NHB males were 210.4 *μ*g/L (mean value), compared to 173.3 *μ*g/L for NHW males. Together, the Pan and Jackson measures were consistent with those reported earlier by Zacharski and colleagues.

With the addition of the GGT values quantified by Pan and Jackson, the differences in SF measures noted in NHANES III provided an additional and potentially important perspective. The cohort of NHB males were reported to have a mean GGT level of 53.9 U/L, compared to a mean of 33.4 U/L for NHW males. Although GGT levels are strongly right-skewed for all populations, the absolute difference in these measures was still very significant. However, we question the validity of a novel approach utilized by Pan and Jackson to explain the observed ethnic differences in these two measures. They also hypothesized that the positive association of age with both serum ferritin and the increased prevalence of inflammatory diseases characterized an aging process. We would suggest as an alternative explanation that the well-established association of inflammatory diseases with aging is primarily due to excess body iron stores and associated greater oxidative stress, as evidenced by significantly higher GGT levels; and both increased body iron stores (as marked by SF) and GGT are dually or synergistically responsible for increased inflammatory diseases.

The interaction of GGT with iron and oxidative stress was first described by Brown et al. [80] in 1998, based on data they obtained from an animal study. Even though glutathione is normally a potent antioxidant, extracellular glutathione can exhibit a prooxidant effect in the presence of GGT [81]. GGT produces cysteinyl glycine as a product of the decomposition of glutathione, and this dipeptide reacts with free iron to induce the Fenton reaction and subsequent production of superoxide, a well-established reactive oxygen species (ROS). Protein phosphatase signal transduction chains respond through cell surface receptors by inducing signaling cascades leading to significant alterations in metabolic activities within the neighboring cells. Researchers noted above significant differences in both GGT and SF measures between African Americans and Caucasian American populations. Similar differences existed when the American NHW male and female populations surveyed during NHANES III were compared to an age- and gender-matched Black African population from Zimbabwe. In 2009, Moyo et al. [82] selected 823 African American men and 557 African American women from NHANES III for the purpose of comparing SF measures with those of 194 Zimbabwean men and 256 Zimbabwean women. The African Americans selected were self-described as non-alcohol-drinking participants of the NHANES III study. The non-alcohol-drinking status was comparable to that described of the Zimbabweans. Serum ferritin measures of the NHB American men and women ranged from 1.78- to 2.05-fold higher than those of their African counterparts in each of three age and gender groupings. Moyo et al. suggested that such different iron measures might have resulted from the American practice, since 1941, of fortifying flour with iron and noted that flour had not been iron-fortified in Zimbabwe. Moyo et al. also noted that more than twenty percent of their African subjects were detected with hepatitis B virus (HBV), hepatitis C virus (HCV), or both. Remarkably, the SF measures of the infected individuals were no higher than those who were uninfected, even though their liver transaminase measures (both ALT and AST) were significantly higher. A second remarkable observation by Moyo et al. was that median GGT levels were 17 U/L among Zimbabwean men, which were less than half the levels reported by Pan and Jackson for the NHB men tested in NHANES III.

Studies in the US and elsewhere have reported that both higher SF and GGT are frequently observed in patients with hepatitis C (HCV). For instance, in 2011, Lambrecht et al. [83] and, earlier, Bonkovsky et al. [84], based on data from a large American trial, the HALT-C Trial, found that increased body iron stores predicted adverse clinical outcomes, including hepatocellular carcinoma and poor response to pharmaceutical treatment. In 2013, Everhart and Wright [85] reported their findings on GGT measures, which were also gathered from the HALT-C Trial participants. Interestingly, except for hepatocellular carcinoma, the outcome measures noted were virtually the same as reported by Lambrecht and colleagues relative to elevated serum SF and iron stores. Although Moyo et al. did not report the degree of infection severity in their African, hepatic-virus-infected subjects, they did report the serum GGT levels of seven men with elevated SF (median levels of 338 *μ*g/L). Among these seven men, median GGT levels were 27 U/L, which was about half the level reported by Pan and Jackson for American NHB men; and only 6 percent of these men were described as HCV-infected. These levels were also much lower than the median GGT level of 115 U/L reported by Everhart and Wright for their HCV-infected (male and female) subjects in the American trial. Hence, it appears that, at the population level, GGT is elevated in the US, compared to Zimbabwe.

However, it must be pointed out that the field work both in the US and in Africa that provided the GGT and SF levels for NHANES III participants and for the African (Zimbabwean) populations described above by Zacharski et al., Pan and Jackson, and Moyo et al. was obtained over no more than a seven-year period between approximately 1988 and 1994. Based on data from a much more recent trial conducted in Africa, Lemoine et al. [86] investigated 135 consecutive treatment-naïve patients diagnosed with chronic HBV. The trial was aimed at evaluating an inexpensive option for a noninvasive test to estimate degrees of liver fibrosis or cirrhosis in HBV-affected patients. The trial was conducted in response to a stated need to find such a test prior to the administration of a drug protocol that had been recently approved by the World Health Organization (WHO) [87]. Based on earlier findings identifying GGT as an independent marker of significant fibrosis [88, 89], the investigators measured Gambian patient GGT levels and platelet counts. The results were matched with patient liver histologies obtained by biopsy. Finally, those results were compared to those obtained from French and Senegalese verification cohorts. The investigation and comparisons found both serum measures correlated very well with liver fibrosis stages. GGT values correlated positively with Metavir (fibrosis) scores (Spearman's correlation coefficient *r* = 0.48,* P* < 0.0001), and platelet count was negatively correlated (*r* = 0.53,* P* < 0.0001). Using the area under the receiver operating characteristics curve (AUROC) for predicting fibrosis score, the synergy of using both measures increased predictability of the tests from using GGT alone (0.77, 95% CI 0.68 to 0.85,* P* = 0.07) or platelet count alone (0.70, 95% CI 0.61 to 0.79,* P* = 0.02), to (0.80, 95% CI 0.72 to 0.88).

Since NHANES III (1988–1994), only a few studies of African Americans in which SF or GGT were obtained have been published. However the SF of 25,435 African Americans (described in the study as Blacks) was measured in the Hemochromatosis and Iron Overload Screening Study (HEIRS). Black male and female SF levels were reported by Acton et al. [90] in connection with their study of self-reported diabetes among HEIRS participants. HEIRS participants included smaller cohorts of American-Hispanic, Asian, Pacific Islander, and Native American, as well as a larger cohort of 43,986 men and women described as Whites. The prevalence of self-described diabetes was 17.48 percent among Blacks. By comparison, the percentages of self-described diabetes among Whites and Native Americans were 11.50% and 16.50%, respectively. The SF (reported as geometric mean levels) among Black men was 231 *μ*g/L, both for those with and for those without diabetes; for Black women, SF levels among women with self-reported diabetes were 113 *μ*g/L and were 85 *μ*g/L among those not reporting diabetes. We described the SF findings for Black participants from NHANES III earlier in reference to study by Zacharski et al. [76]. Also described as geometric means, male Black SF measures from NHANES III, measured approximately 10 years earlier than those obtained for HEIRS, appear to be at least 60 *μ*g/L higher in the more recent study. A proportionally similar SF increase of about 50 *μ*g/L appears to have occurred among the White men measured in HEIRS. Hence, SF levels appear to be rising in the US population over time.

Since both NHANES III and HEIRS were structured as nationally representative cohorts and had very similar age distributions, we suspect the temporal increases in SF measures among both Black and White men might have resulted from the same types of pressures that caused the steep increases of serum GGT over a similar time frame as found among Korean steel plant employees in the study described below by Lee and colleagues [97]. We are familiar with only one other large American study that contained a large cohort of African Americans in which SF (but not GGT) was reported. These data were published in a study by Olesnevich et al. [91] in 2012, with an accompanying corrigendum [92]. Referred to as the HANDLS study, the investigation measured SF and multiple other factors in subjects from a low-income urban population in the inner-city of Baltimore. SF data, from both African American and White male and female participants, was collected over a nearly five-year period with a mean collection date sometime in early 2006. The aim of Olesnevich and her colleagues was to evaluate the risk of subjects developing CHD relative to their SF measures. The investigators developed an analysis using Framingham Heart Study's 10-year risk algorithm. Based on this analysis, they determined that, for HANDLS participants, elevated SF levels were associated with increased 10-year CHD risk, independent of elevated C-reactive protein levels. They noted in their finding that “SF is a significant predictor of 10-year hard CHD risk for HANDLS participants” and that the correlation of CHD risk with SF levels was consistent with the effect first hypothesized in 1981 by Sullivan and discussed and cited herein [77, 78].

The absolute height of the SF levels measured in Baltimore, as well as in the large HEIRS study reported above by Acton et al. [90], raises concerns, as they do for even higher SF levels in other areas of the world, notably China [93, 94]. In an information statement published by the World Health Organization (WHO) in 2011 [95], it was stated that SF above 300 *μ*g/L in men and 200 *μ*g/L in women could be considered a warning of “severe risk of iron overload.” In the 2008 above-reported study from China by Sun et al. [94], these risk benchmarks were surpassed at SF levels measured in the third quartiles among both male and female populations.

## 8. GGT: Chronic Kidney Disease and Albuminuria 

In a previous section, we described incident obesity, MetS, prehypertension, and insulin resistance relative to study-entry GGT levels for several large cohorts of Korean men. In another large study from Korea, Ryu et al. [96] followed up on 10,337 healthy men with normal blood pressure and without diabetes for 30 months to ascertain incident chronic kidney disease (CKD). In their 2007-reported study, the authors found that 266 men had progressed to a diagnosis of CKD during the relatively short observation period and that a trend toward incident CKD started in the second quartile GGT levels (above 19 U/L) and became significant for subjects with fourth quartile GGT over 40 U/L. The HR for incident CKD when GGT was above 40 U/L was 1.71 (95% CI; 1.22–2.39),* P* for trend <0.001. Ryu et al. had noted that, in 1999, the US reported a persistent increase in kidney failure requiring either dialysis therapy or kidney transplantation and that similar trends were taking place in Korea. They reported data from a 2004 report (in Korean) by the Korean Society of Nephrology, Registry Committee, that the prevalence of End-Stage Renal Disease (ESRD) patients that required renal replacement therapy had more than doubled between 1994 and 2004. Interestingly, and likely relevant to the work of Ryu and colleagues, Lee et al. [97] reported findings from a longitudinal study of 8,082 men, all personnel of a Korean steel plant, that a strong upward trend in GGT levels was reported during the period from 1996 to 2003. Lee et al. reported that GGT was measured annually and increased by nearly twofold over seven years; geometric mean values of serum GGT were measured to be 10.5, 14.5, 18.6, 20.2, 21.4, 22.1, 26.6, and 29.2 U/L, in successive years of the study. This population level, consistent upward, trend is alarming, especially considering that ESRD is rising in parallel. This trend toward increasing GGT levels was noted in Section 3 in the comparison of relatively much lower GGT levels found in participants of the Framingham Offspring Study measured between 1978 and 1982 to the participants of the plant-based food spreads cholesterol trial conducted in Netherlands around the year 2000. Interestingly, but equally alarming, the annual increases in GGT levels in this cohort of Korean men were approximately the same as that which occurred among Dutch men and women around that same year. A possible consequence of this ominous temporal trend appears to be reflected in a very recent report of increased GGT levels as a predictor of mortality in Korean ESRD patients undergoing peritoneal dialysis (PD). In 2015, Park et al. [98] reported the result of an observational study of a subcohort of 820 patients with GGT levels measured starting in 2009. As reported in many other studies, serum ferritin (SF) levels correlated both with GGT and with mortality. Patients were followed up for a median of 34 months, during which there were 116 deaths. CVD was the leading cause of death; it was followed closely by infectious diseases; together, these two causes accounted for approximately two-thirds of all mortalities. Consistent with an observed “obesity paradox” in this study, BMI was negatively associated with mortality (*β* = −0.91, *P* = 0.027).

In a recent population-based, cross-sectional study reported from China, Sun et al. [99] reviewed multiple biomarkers, lifestyle factors, and clinical characteristics of 9,702 males and females aged 40 years and older. Their 2014-reported study was aimed to evaluate the relationship of serum GGT with the prevalence of low-grade albuminuria and increased urinary albumin excretion. The prevalence of each condition was found to be 23.4 percent and 6.6 percent, respectively. The investigators noted that albuminuria was also a marker of endothelial dysfunction. After analyzing the subject information, they observed that individuals with either low-grade albuminuria or urinary albumin excretion had significantly higher GGT levels (all *P* < 0.05), when compared to subjects with normal albumin excretion. For both genders, the prevalence of albuminuria increased from the lowest to the highest GGT quartiles (all* P* for trend <0.0001). Above-median GGT levels were correlated with increased odds of low-grade albuminuria and urinary albumin excretion (all* P* for trend <0.0001). An interesting observation from this study is that the mean BMI of the participants was 23.7 kg/m^2^ and that no increased risk of low-grade albuminuria or urinary albumin excretion was found among individuals having a BMI over 28 kg/m^2^, the level considered to mark obesity by Chinese standards. This is interesting because BMI correlated positively across all GGT quartiles. However, since only 697 of 9,702 (7.2%) of the study participants were reported to be obese, this could indicate the emergence of an “obesity paradox” for kidney dysfunction in China, similar to what has been observed in Korea. It also might further imply that those subjects classified as overweight (BMI 24–28), who were reported to have the highest GGT-related risk, were likely to have a risk more closely akin to the author's risk model number one, which shows a pattern of decreased glomerular filtration across each of GGT quartiles two, three, and four, when each is compared to the first GGT quartile, *P* < 0.05,* P* for trend <0.0001.

Sun et al. demonstrate an attenuation of risk in their second and third risk models which might have been primarily due to adjustment for BMI in the second model and then additional adjustments in a third risk model for several factors, including blood pressure and HOMA-IR, that, as are demonstrated above, can occur subsequent to, or downstream of, an elevated serum GGT. We suggest that these adjustments might have been overadjustments that perhaps led Sun et al. to have “speculated that elevated serum GGT level might be a biomarker rather than a true causal risk factor for oxidative stress, inflammation and albuminuria.” Sun et al. also found that subjects with diabetes were marginally less likely to reach either of their measured endpoints than those without diabetes. Based on this observation, they also “speculated that subjects diagnosed with diabetes were more inclined to receive further treatment, which may correct their urinary albumin excretion.” Here again, similar to the observation of obesity occurring downstream of elevated GGT, there are a number of studies that show that new onset diabetes often occurs more frequently among individuals with higher GGT levels, irrespective to body weight or BMI.

Examples of these prospective studies include Perry et al. [100], a study of 7,458 nondiabetic men in the U.K. who were followed up for a mean of 12.8 years. As in other prospective GGT studies, the GGT levels of men who became diabetic were significantly higher than those of the men who did not, *P* < 0.0001. They also noted a smooth, graded increase in age-adjusted risk with increasing GGT levels. A second example is found in a study reported from France by Gautier et al. [101]. The nine-year risk of incident diabetes was related to study-entry GGT levels, and very similar to the BMI observation of Sun et al. described above. Individuals having BMI below 27 kg/m^2^ demonstrated significantly greater risk of proceeding to a diagnosis of diabetes when compared to those having higher BMI and GGT in the same range of 20 to 40 U/L. Gautier et al. reported that, in the four risk models used, BMI above 27 kg/m^2^ was not associated with incident diabetes, unless GGT levels were above 40 U/L.

We question the “speculation” of Sun et al. because there are now substantial findings that consistently place the juxtaposition of moderately elevated GGT levels before the onset of other “traditional risk factors” such as obesity, insulin resistance, elevated cholesterol, and glucose measurements. Since onset or observation of these conditions can occur shortly after a rapid rise in GGT levels, the practice of some investigators to adjust for these and other “risk factors” in multivariate models might be at times incorrect. An example of such an incorrect procedure might have affected the data analyses and conclusions of a cross-sectional study of the association of serum GGT with chronic kidney disease that was recently reported in the US by Teppala et al. [102]. In this study, the investigators reviewed the data from NHANES 1999–2002 for 9,516 participants. Even though the investigators cited and discussed the findings of Ryu et al. from Korea, as we did above as well [96], they modeled GGT as an “ordinary variable” and therefore derived the odds of CKD as if GGT had no bearing on other physical and clinical conditions, including BMI, hypertension, and diabetes. Since, by 1999–2002, most of America had already experienced the “obesity epidemic,” [103] which in the US was most robust during the 1980s and 1990s, the BMI levels by the time of this study (1999–2002) had already reached a mean of 27.97 kg/m^2^, a level almost identical to 28 kg/m^2^, the obesity level in China, above which Sun et al. found no risk of impaired kidney function among their small (7.2%) subgroup of subjects found to be obese. Also, Ryu et al. had a similarly small-sized subgroup of Korean subjects with a BMI over 28 kg/m^2^. It is notable that, in the American cross-sectional study, Teppala et al. found no risk associated with higher serum GGT levels. We suggest that this was related to the much greater numbers of participants (approximately half) having BMI measures over 28 kg/m^2^, and at that level or above, transition to disease end points appears to be muted or dampened.

An interesting observation relevant to a number of population studies that we have reviewed is apparent in both the Ryu et al. and Sun et al. studies. The trends in study population characteristics were virtually identical for all of the many common characteristics described in each of these studies. For example, the following characteristics were described in each study as correlating positively across each GGT level quartile: BMI, both systolic and diastolic blood pressure, LDL-cholesterol, total cholesterol, triglycerides, fasting plasma glucose, and HOMA-IR (insulin resistance); and one measure that correlated inversely across all GGT quartiles was GFR or (eGFR) for glomerular filtration, a measure of kidney function. For all measures, all* P* trends were <0.0001.

## 9. Conclusion

In this paper, we have presented a review of the serum marker, GGT, and we have shown that it has remarkable predictive powers for a multitude of diseases and chronic conditions whose incidence is on the rise across the industrialized world. These include, in addition to liver disease and bile duct disorders, cardiovascular disease, metabolic syndrome, obesity, type-II diabetes, gestational diabetes, hypertension, heart failure, cancer, and all-cause mortality. We note that the population-wide level of GGT has been steadily increasing over time in the last three decades in the US and two decades in Korea. Several studies indicate this upward trend has affected other populations, including Europe, as well. We suspect this may be indicative of increased exposure to environmental xenobiotics, especially POPs, as well as increased body iron burden. Serum ferritin levels closely parallel serum GGT levels, further implicating iron overload as a factor. This may be attributed, in part, to the practice in America of fortifying flour with iron and with folate, which promotes iron uptake.

It is also noteworthy that Black males in America have particularly high GGT levels that are directly linked to increased risk of multiple chronic diseases. A comparison between Black American males and Black males in Zimbabwe revealed an average GGT level among the Americans that was more than double that found in the Zimbabweans. A factor here may be the lack of iron fortification in flour in Zimbabwe. An important question to ask is to what extent this measure is pervasive throughout the United States and the world today. If so, it predicts even greater disease prevalence in the future.

The prevalence of several other clinical symptoms are correlated with GGT, including hypertension, insulin resistance, artery calcification, and albuminuria, as well as biological markers including lipids, creatine, triglycerides, uric acid, HbA1c, and hs-CRP. In many cases, GGT is a stronger predictor of disease risk than these other symptoms and markers.

Low antioxidant defenses are also correlated with elevated GGT, particularly reduced levels of glutathione. GGT is needed to metabolize glutathionylated xenobiotics in the liver and multiple other tissue sites including the lungs, and this is a simple explanation for its elevation in association with increased exposure to xenobiotics. GGT induces oxidative stress in the artery wall in the presence of free iron, and GGT also likely is an indicator of depleted supply of glutathione, especially in the liver, which leads to a cascade of problems related to increased oxidative stress.

We propose that simple lifestyle changes to, when possible, avoid toxic chemical exposure and limit iron intake may have great benefit in alleviating the burden of GGT-related chronic diseases. Dietary changes targeted at improving red blood cell membranes might prove to be a life-saving strategy for many. New studies are needed to ascertain if the novel finding demonstrated herein for US Navy bottlenose dolphins translates to improved human health, lower GGT and serum ferritin levels, and protection from disease. Frequent blood donation or therapeutic phlebotomy is a relatively low-risk strategy for reducing body iron burdens. Curcumin, having a proven capability of chelating poorly liganded iron, has been shown to ameliorate many diseases linked to elevated GGT. For individuals affected by iron overload from multiple blood transfusions, chemical iron chelation has been demonstrated to maintain GGT at low-risk levels and reduce toxic levels of free iron in the blood. Pregnant women should be monitored for GGT and/or SF levels and encouraged to reduce iron intake if levels are elevated. It should be common practice in medicine to routinely measure GGT, as it is a significantly better predictor of disease than serum lipids, which are already routinely measured.
